# Supplementary figures and images for: Metabolic response of Scapharca subcrenata to heat stress using GC/MS-based metabolomics
Source: PeerJ. 2020 Jan 28;8:e8445. doi: 10.7717/peerj.8445 (PMC6993748; doi:10.7717/peerj.8445)

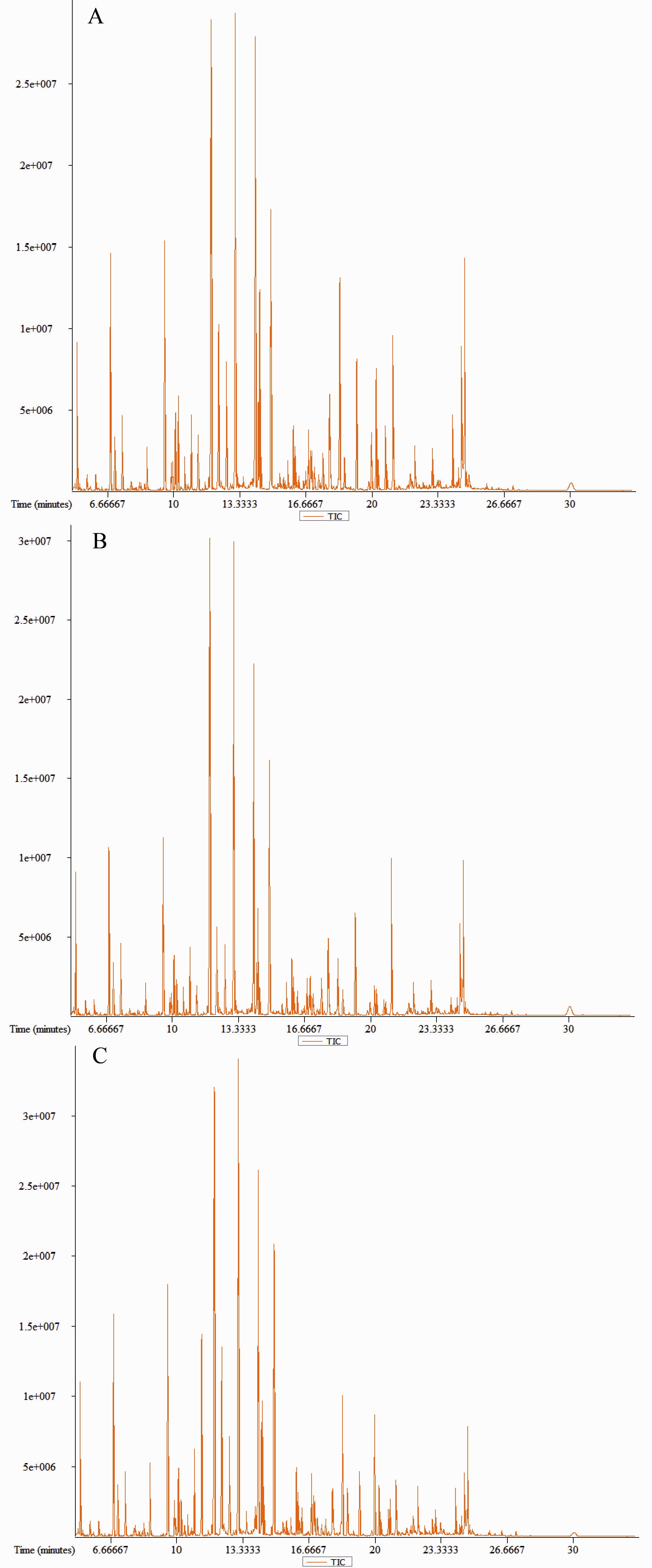

Supplement: Supplemental Information 1 — (A): the control (B): 2 h (C): 24 h. The ordinate shows the relative mass abundance, and the abscissa shows the retention time. [file peerj-08-8445-s001.jpg]

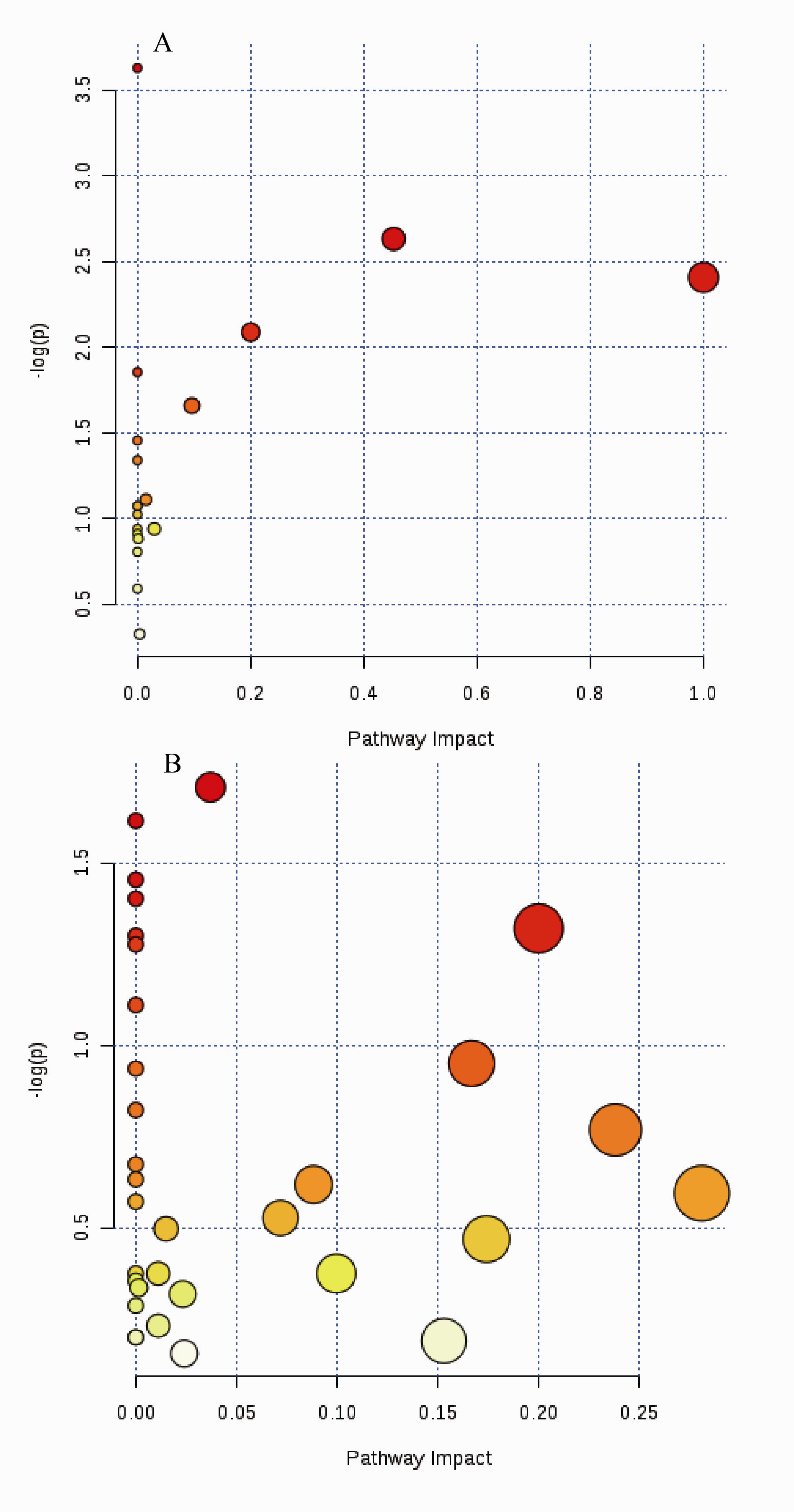

Supplement: Supplemental Information 2 — A: 2 h, B: 24 h. [file peerj-08-8445-s002.jpg]
