# Supplementary material for: Metabolic response of Scapharca subcrenata to heat stress using GC/MS-based metabolomics
Source: PeerJ. 2020 Jan 28;8:e8445. doi: 10.7717/peerj.8445 (PMC6993748; doi:10.7717/peerj.8445)
Supplement: Supplemental Information 3 [file peerj-08-8445-s003.docx]

| **Metabolites** | **VIP value** | ***P* value** | **Fold Change** |
| --- | --- | --- | --- |
| Glutaric acid | 2.22 | 0.0001 | 0.56 |
| Biphenyl | 2.28 | 0.0002 | 1.79 |
| Azelaic acid | 2.24 | 0.0003 | 0.55 |
| Glutamic acid | 2.24 | 0.0003 | 0.74 |
| Lactitol | 2.30 | 0.0004 | 0.45 |
| Sophorose | 2.07 | 0.0004 | 1.36 |
| Glucose-6-phosphate | 2.18 | 0.0005 | 0.45 |
| 6-hydroxy caproic acid dimer | 2.18 | 0.0007 | 0.58 |
| Quinolinic acid | 2.10 | 0.0009 | 1.66 |
| Carbazole | 2.13 | 0.0009 | 2.01 |
| Aspartic acid | 2.14 | 0.0010 | 0.77 |
| Adipic acid | 2.15 | 0.0011 | 0.72 |
| O-phosphorylethanolamine | 2.15 | 0.0018 | 1.60 |
| 1,5-anhydroglucitol | 1.91 | 0.0021 | 1.34 |
| 3-indoleacetonitrile | 2.03 | 0.0024 | 0.00 |
| Cyclohexylsulfamic acid | 2.03 | 0.0026 | 0.15 |
| 1-hexadecanol | 2.00 | 0.0033 | 1.60 |
| Menthone | 1.93 | 0.0038 | 1.51 |
| Erythrose | 1.93 | 0.0065 | 1.31 |
| Taurine | 1.99 | 0.0073 | 1.29 |
| Glucosaminic acid | 1.84 | 0.0118 | 0.33 |
| 2-hydroxypyridine | 1.79 | 0.0121 | 1.27 |
| Cytosine | 1.70 | 0.0144 | 1.48 |
| Alpha-Tocopherol | 1.80 | 0.0154 | 1.49 |
| 2,3-dihydroxypyridine | 1.78 | 0.0157 | 1.40 |
| M-cresol | 1.71 | 0.0180 | 1.35 |
| Oleic acid | 1.73 | 0.0184 | 0.70 |
| Creatine degr | 1.71 | 0.0200 | 1.28 |
| Adenine | 1.77 | 0.0200 | 1.61 |
| Mevalonic acid lactone | 1.64 | 0.0217 | 1.24 |
| Nicotinoylglycine | 1.66 | 0.0218 | 1.22 |
| 4-Hydroxymethyl-3-methoxyphenoxyacetic acid | 1.69 | 0.0235 | 1.19 |
| Diglycerol | 1.63 | 0.0269 | 0.45 |
| 1-hydroxyanthraquinone | 1.56 | 0.0271 | 1.26 |
| Prostaglandin E2 | 1.57 | 0.0273 | 1.27 |
| 4-methylumbelliferone | 1.57 | 0.0332 | 1.27 |
| 1-methylhydantoin | 1.50 | 0.0373 | 1.23 |
| Carbobenzyloxy-L-leucine deg | 1.50 | 0.0380 | 1.34 |
| Lactic acid | 1.39 | 0.0400 | 1.28 |
