# Supplementary material for: Metabolic response of Scapharca subcrenata to heat stress using GC/MS-based metabolomics
Source: PeerJ. 2020 Jan 28;8:e8445. doi: 10.7717/peerj.8445 (PMC6993748; doi:10.7717/peerj.8445)
Supplement: Supplemental Information 4 [file peerj-08-8445-s004.docx]

| **Metabolites** | **VIP value** | ***P* value** | **Fold Change** |
| --- | --- | --- | --- |
| Lactitol | 2.10 | 0.0000 | 0.00 |
| Glutaric acid | 2.04 | 0.0000 | 0.21 |
| Azelaic acid | 2.03 | 0.0000 | 0.32 |
| Oxamic acid | 2.00 | 0.0000 | 0.66 |
| 6-hydroxy caproic acid dimer | 2.00 | 0.0000 | 0.33 |
| Biphenyl | 1.99 | 0.0000 | 2.13 |
| Adipic acid | 1.98 | 0.0000 | 0.32 |
| Quinolinic acid | 1.96 | 0.0000 | 2.20 |
| Oleic acid | 1.93 | 0.0000 | 0.48 |
| Tartaric acid | 1.90 | 0.0001 | 0.35 |
| Elaidic acid | 1.85 | 0.0002 | 0.54 |
| Histidine | 1.81 | 0.0004 | 0.51 |
| Taurine | 1.79 | 0.0004 | 1.42 |
| Phloroglucinol | 1.78 | 0.0009 | 2.24 |
| Cyclohexylsulfamic acid | 1.75 | 0.0009 | 0.06 |
| Nicotinoylglycine | 1.73 | 0.0012 | 1.32 |
| 1-methylhydantoin | 1.74 | 0.0013 | 1.39 |
| 5-methoxytryptamine | 1.73 | 0.0014 | 1.35 |
| 1-hexadecanol | 1.72 | 0.0015 | 1.79 |
| Cis-gondoic acid | 1.72 | 0.0016 | 0.54 |
| D-talose | 1.71 | 0.0018 | 1.95 |
| 2-Amino-3-methyl-1-butanol | 1.70 | 0.0020 | 1.33 |
| Gallic acid | 1.67 | 0.0022 | 1.23 |
| 3-indoleacetonitrile | 1.67 | 0.0024 | 0.00 |
| Glycerol | 1.68 | 0.0024 | 0.82 |
| O-phosphorylethanolamine | 1.64 | 0.0029 | 1.76 |
| Erythrose | 1.65 | 0.0029 | 1.31 |
| Glucose-6-phosphate | 1.62 | 0.0034 | 0.37 |
| Pimelic acid | 1.62 | 0.0037 | 0.14 |
| Methionine sulfoxide | 1.61 | 0.0038 | 5.28 |
| Glycolic acid | 1.62 | 0.0039 | 0.84 |
| Oxalic acid | 1.61 | 0.0046 | 4.22 |
| Alanine | 1.60 | 0.0048 | 0.77 |
| 2'-Deoxycytidine 5'-triphosphate degr prod | 1.58 | 0.0055 | 1.26 |
| Pyruvic acid | 1.60 | 0.0055 | 1.45 |
| Mevalonic acid lactone | 1.58 | 0.0057 | 1.38 |
| Analyte 138 | 1.56 | 0.0060 | 1.88 |
| (+-)-Dihydrocarveol | 1.57 | 0.0067 | 0.44 |
| Palmitic acid | 1.55 | 0.0069 | 0.72 |
| Glucosaminic acid | 1.57 | 0.0071 | 0.22 |
| 2,3-dihydroxypyridine | 1.53 | 0.0071 | 1.53 |
| O-phosphonothreonine | 1.56 | 0.0074 | 0.59 |
| P-benzoquinone | 1.54 | 0.0082 | 1.27 |
| Hydroxylamine | 1.52 | 0.0086 | 1.28 |
| 3-hydroxynorvaline | 1.50 | 0.0100 | 1.31 |
| Methyl hexadecanoate | 1.50 | 0.0107 | 0.00 |
| Menthone | 1.47 | 0.0109 | 1.55 |
| 3-aminopropionitrile | 1.50 | 0.0111 | 1.26 |
| 4-hydroxybutyrate | 1.51 | 0.0115 | 0.00 |
| Hypoxanthine | 1.50 | 0.0122 | 0.24 |
| Prostaglandin E2 | 1.46 | 0.0133 | 1.25 |
| Terephthalic acid | 1.46 | 0.0139 | / |
| 3-(1-Pyrazolyl)-L-alanine | 1.47 | 0.0147 | 0.14 |
| D-(glycerol 1-phosphate) | 1.43 | 0.0156 | 1.31 |
| Tryptophan | 1.42 | 0.0160 | 0.65 |
| Phthalic acid | 1.46 | 0.0160 | 0.00 |
| Adenosine | 1.43 | 0.0171 | 1.63 |
| Thymidine 5'-monophosphate degr prod | 1.42 | 0.0172 | 0.23 |
| Thymidine | 1.43 | 0.0173 | 0.16 |
| Cytidine-monophosphate degr prod | 1.41 | 0.0192 | / |
| 2-hydroxyquinoline | 1.41 | 0.0198 | 0.48 |
| Dibenzofuran | 1.40 | 0.0202 | 1.20 |
| Diglycerol | 1.38 | 0.0202 | 0.46 |
| 3-hydroxybutyric acid | 1.40 | 0.0203 | 0.05 |
| Hydrocinnamic acid | 1.39 | 0.0214 | 1.25 |
| Palmitoleic acid | 1.38 | 0.0215 | 0.55 |
| Spermidine | 1.37 | 0.0247 | 1.65 |
| Thymine | 1.37 | 0.0252 | 1.40 |
| 2-aminophenol | 1.38 | 0.0254 | 0.20 |
| Scopoletin | 1.35 | 0.0258 | 1.23 |
| Pentadecanoic acid | 1.31 | 0.0274 | 0.77 |
| Phenyl beta-D-glucopyranoside | 1.35 | 0.0288 | 0.00 |
| Alpha-Tocopherol | 1.32 | 0.0312 | 1.40 |
| 5-Hydroxyindole-2-carboxylic acid | 1.34 | 0.0312 | 0.79 |
| 2-hydroxypyridine | 1.29 | 0.0323 | 1.22 |
| Beta-Glycerophosphoric acid | 1.29 | 0.0324 | 1.31 |
| Guaiacol | 1.33 | 0.0326 | 0.60 |
| Benzoic acid | 1.31 | 0.0334 | 1.18 |
| Cytidine-monophosphate | 1.31 | 0.0338 | 1.24 |
| Beta-hydroxypyruvate | 1.28 | 0.0350 | 1.16 |
| Mono(2-ethylhexyl)phthalate | 1.30 | 0.0366 | 1.24 |
| Carbazole | 1.25 | 0.0367 | 2.14 |
| L-allothreonine | 1.26 | 0.0390 | 1.98 |
| N-Methyl-L-glutamic acid | 1.25 | 0.0395 | 1.26 |
| 4-Hydroxycyclohexanecarboxylic acid | 1.29 | 0.0396 | 3.26 |
| 4-Vinylphenol dimer | 1.24 | 0.0406 | 1.50 |
| Acetylsalicylic acid | 1.24 | 0.0449 | 0.17 |
| 1-hydroxyanthraquinone | 1.22 | 0.0473 | 1.22 |
| Hippuric acid | 1.24 | 0.0476 | 3.16 |
| Creatine degr | 1.21 | 0.0491 | 1.25 |
