# Supplementary material for: Metabolic response of Scapharca subcrenata to heat stress using GC/MS-based metabolomics
Source: PeerJ. 2020 Jan 28;8:e8445. doi: 10.7717/peerj.8445 (PMC6993748; doi:10.7717/peerj.8445)
Supplement: Supplemental Information 6 [file peerj-08-8445-s006.docx]

| **Pathway** | ***P*** | **-log(*P*)** | **Impact** |
| --- | --- | --- | --- |
| Glutathione metabolism | 0.003890 | 5.5495 | 0.0519 |
| Histidine metabolism | 0.004892 | 5.3202 | 0.2381 |
| beta-Alanine metabolism | 0.007260 | 4.9254 | 0.0 |
| Nitrogen metabolism | 0.021302 | 3.849 | 0.0 |
| Alanine, aspartate and glutamate metabolism | 0.022735 | 3.7838 | 0.4525 |
| Aminoacyl-tRNA biosynthesis | 0.027226 | 3.6036 | 0.0 |
| Nicotinate and nicotinamide metabolism | 0.049618 | 3.0034 | 0.0 |
| Pantothenate and CoA biosynthesis | 0.056327 | 2.8766 | 0.0 |
| Ubiquinone and other terpenoid-quinone biosynthesis | 0.07644 | 2.5712 | 0.0 |
| Glyoxylate and dicarboxylate metabolism | 0.07818 | 2.5487 | 0.0370 |
| Glycerolipid metabolism | 0.07818 | 2.5487 | 0.3857 |
| Arginine and proline metabolism | 0.099002 | 2.3126 | 0.1202 |
| Butanoate metabolism | 0.11067 | 2.2012 | 0.0 |
| Synthesis and degradation of ketone bodies | 0.12421 | 2.0857 | 0.0 |
| D-Glutamine and D-glutamate metabolism | 0.12421 | 2.0857 | 1.0 |

|  |  |  |
| --- | --- | --- |
|  |  |  |
|  |  |  |
|  |  |  |
|  |  |  |
|  |  |  |
|  |  |  |
|  |  |  |
|  |  |  |
|  |  |  |
|  |  |  |
|  |  |  |
|  |  |  |
|  |  |  |
|  |  |  |
